# Molecular characterization of marine and coastal fishes of Bangladesh through DNA barcodes

**DOI:** 10.1002/ece3.7355

**Published:** 2021-04-07

**Authors:** Md. Sagir Ahmed, Sujan Kumar Datta, Tonmoy Saha, Zarif Hossain

**Affiliations:** ^1^ Department of Zoology University of Dhaka Dhaka Bangladesh; ^2^ Department of Zoology Jagannath University Dhaka Bangladesh; ^3^ Department of Oceanography University of Dhaka Dhaka Bangladesh

**Keywords:** actinopterygii, barcoding, COI, elasmobranchii, genetic diversity, phylogeny

## Abstract

This study describes the molecular characterization of marine and coastal fishes of Bangladesh based on the mitochondrial cytochrome c oxidase subunit I (COI) gene as a marker. A total of 376 mitochondrial COI barcode sequences were obtained from 185 species belonging to 146 genera, 74 families, 21 orders, and two classes of fishes. The mean length of the sequences was 652 base pairs. In Elasmobranchii (Sharks and rays), the average Kimura two parameter (K2P) distances within species, genera, families, and orders were 1.20%, 6.07%, 11.08%, and 14.68%, respectively, and for Actinopterygii, the average K2P distances within species, genera, families, and orders were 0.40%, 6.36%, 14.10%, and 24.07%, respectively. The mean interspecies distance was 16‐fold higher than the mean intraspecies distance. The K2P neighbor‐joining (NJ) trees based on the sequences generally clustered species in accordance with their taxonomic position. A total of 21 species were newly recorded in Bangladesh. High efficiency and fidelity in species identification and discrimination were demonstrated in the present study by DNA barcoding, and we conclude that COI sequencing can be used as an authentic identification marker for Bangladesh marine fish species.

## Graphical abstract

This study attempted to molecular characterization of marine and coastal fishes of Bangladesh based on COI gene as a marker. A total of 376 mitochondrial COI barcode sequences were obtained from 185 species belonging to 146 genera, 74 families, 20 orders, and two classes of fishes. Our barcode data can define the diversity of marine fishes across genera, families, and orders. A total of 21 species were newly recorded in Bangladesh. This is the first comprehensive barcode reference library for marine and coastal fishes of Bangladesh.

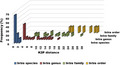

## 1 INTRODUCTION

Bangladesh has vast coastal and marine resources along its south edge as the Bay of Bengal is situated in the south of Bangladesh. There are a total of 166,000 square km water areas including the Exclusive Economic Zone (EEZ) which is larger than the country's total land area of 147,500 square km. The country is rich not only in terms of its vast water areas but also in terms of biological diversity. The marine fisheries sector plays an important role in the economy of Bangladesh in terms of nutrition, income, employment, and foreign exchange earnings (DoF, 2018). Fish provides about 60% of animal protein in the daily dietary requirement of 160 million people of the country. Marine fisheries alone contribute 654,687 metric tons which is 15.31% of the country's total fish production (DoF, 2018).

Nonetheless, description and information of marine fishes of Bangladesh are scattered throughout a wide range of scientific publications (Hussain, 1971; Shafi & Quddus, 1982; Rahman et al., 1995, 2009). Estimates of total fish species vary from 170 (Shafi & Quddus, 1982) to 402 (Rahman et al., 2009), 442 (IUCN, 2000), or 475 (Hussain, 1971) including the migratory and estuarine species. The diversity of marine fish species in the neighboring countries of India and Myanmar were recorded as 2,443 and 600, respectively (Froese & Pauly, 2019; Gopi & Mishra, 2015). This indicates that the diversity of the marine and coastal fishes of Bangladesh is poorly recorded. It is evident from the published articles, books, and review papers on Bangladeshi marine fishes (Ahmed et al., 2019; Hussain, 1971; IUCN, 2000; Rahman et al., 2009; Shafi & Quddus, 1982) that the ichthyofaunal diversity statistics are incomplete. Moreover, till date, no molecular taxonomic study has been undertaken on the marine and coastal ichthyofaunal diversity of Bangladesh.

The accurate identification of fish species is a pivotal component to protect the extant ichthyofaunal biodiversity and to perform regular assessments of local fish faunas for conservation planning (Ahmed et al., 2019). Currently, partial cytochrome c oxidase subunit I (COI) sequences (DNA barcodes) are applied extensively as a complement to the traditional morpho‐taxonomy for standardized and regular species identification (Chin et al., 2016; Filonzi et al., 2010; Hebert et al. 2003; Shehata et al., 2019). The marked divergence and lack of overlap between intraspecific and interspecific genetic distances is the primary reason for the selection of COI as the standard barcode gene (Hebert et al., 2003). More importantly, COI evolution is sufficiently rapid to allow the discrimination of very closely related species in most groups, as well as taxonomically significant intraspecific variation associated with geographic structure (Bucklin et al., 2011).

DNA barcoding has been successfully identified marine ichthyofauna and provided the wealth of DNA barcode information in many places, such as Australia (Ward et al., 2005), Canada (Hubert et al., 2008; Steinke et al., 2009), India (Lakra et al., 2011), China (Wang et al., 2018; Zhang, 2011; Zhang & Hanner, 2012), Portugal (Costa et al., 2012), Germany (Knebelsberger et al., 2014), Taiwan (Bingpeng et al., 2018; Chang et al., 2017), Vietnam (Thu et al., 2019), and Indonesia (Limmon et al., 2020).

Molecular‐based approach becomes a necessary tool when there are difficulties in morphologically similar groups or damaged samples which is a challenge even if experts are available. As a highly overpopulated country, anthropogenic activities, overfishing, habitat destruction, and natural disasters have generated significant impacts on the biodiversity and structure of the fish community in Bangladesh. Unfortunately, the marine ichthyofauna of Bangladesh remains unexplored due to the lack of taxonomists. Hence, adopting an authentic and quick identification method is essential to assist fishery managers, scientists, and policymakers for sustainable management of these invaluable marine resources. This study aims to build a DNA‐based barcode library of the morphologically identified marine and coastal fish species of Bangladesh using partial COI gene sequence.

## 2 MATERIALS AND METHODS

### 2.1 Study area and specimen collection

**FIGURE 1:**
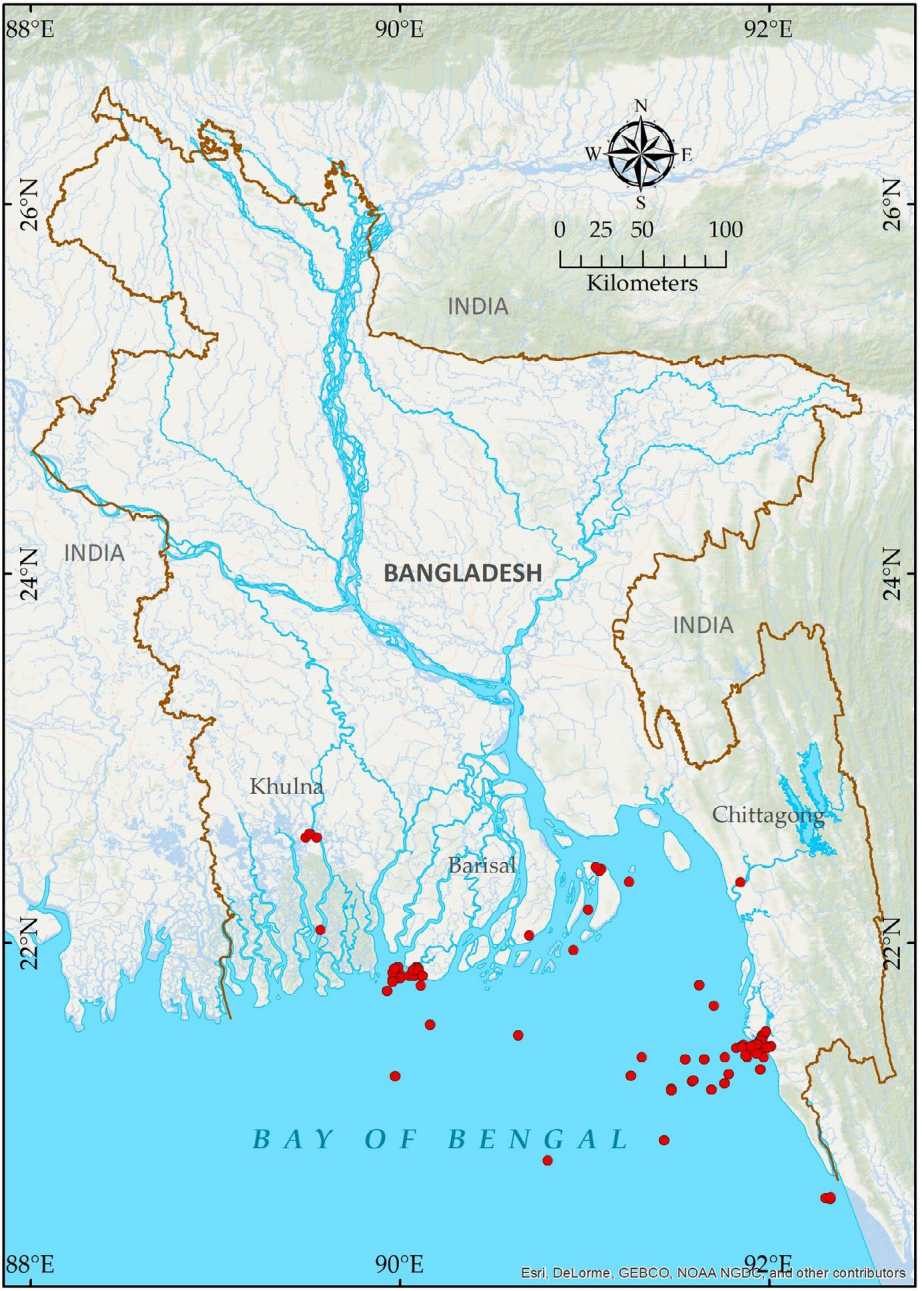
Map of the specimens collecting sites in Bangladesh. A symbol may cover more than one collecting site

Fish samples were collected from marine and coastal habitats, fish landing centers, fish markets or from the local fishermen from July 2015 to June 2019 (Figure 1). Most of the specimens were collected from the Cox's Bazar and Patuakhali regions. At least three specimens were collected for each species. In case of rare ones, only a single specimen was analyzed. Personal fishing was also conducted to collect some rare and noncommercial fish species whenever necessary. Digital photographs of all the fishes were taken immediately and taxonomic identification of specimens was done following previous reports (Talwar & Jhingran, 1991; Carpenter & De Angelis, 2002; Carpenter & Niem, 2002; Last et al., 2016; Nakabo, 2002; Rahman et al., 2009; Siddiqui et al., 2007). Immediately after collecting the specimens, tissue samples were excised and stored in 90% ethanol. Voucher specimens were fixed with 10% formalin and then transferred to 70% ethanol solution for preservation. Voucher specimens were transported to Dhaka and deposited in the Dhaka University Zoology Museum (DUZM).

### 2.2 DNA barcoding

DNA was isolated from muscle sample using the QIAGEN DNeasy Blood & Tissue Kit following the manufacturer's protocol, under sterile condition. The concentration of the isolated DNA was measured in NanoDrop™ spectrophotometer to evaluate its quality and quantity. A 658 bp long fragment from the 5′ region of the COI gene was PCR‐amplified by the primer pair FishF2 5′TCGACTAATCATAAAGATATCGGCAC3′ and FishR2 5′ACTTCAGGGTGACCGAAGAATCAGAA3′. The primer pair FishF1 5′TCAACCAACCACAAAGACATTGGCAC3′ and FishR1 5′TAGACTTCTGGGTGGCCAAAGAATCA3′ were used for the amplification of COI that failed to amplify using FishF2/FishR2 (Ward et al., 2005). The PCR amplification of each sample was conducted in a 25 µl volume comprised of 23 µl of PCR Master Mix and 2 µl of template DNA that was subsequently spun for 30 s for homogenization. The components of the PCR Master Mix were as such: 12.5 µl Taq Polymerase, 8.5 µl Nano Pure water, 1 µl forward primer, and 1 µl reverse primer. The PCR amplification was carried out in the ASTEC Thermal Cycler GeneAtlas (Astec Co. Ltd.) where the thermocycling profile was customized as such: an initial denaturation at 95℃ for 5 min followed by 41 cycles of denaturation at 95℃ for 30 s, primer annealing at 54℃ for 30 s, extension for 72℃ for 1 min, and a final extension at 72℃ for 5 min. The PCR products were kept at room temperature for 15 min, and preserved afterward at −20℃ until further downstream application. The amplicons were further identified through electrophoresis in a 1% agarose gel. Then they were purified using PureLink™ PCR purification kit. The good quality amplicons were then parceled for sequencing to the First BASE laboratories, Malaysia where Sanger dideoxy sequencing was conducted using ABI PRISM 3730xl Genetic Analyzer exploiting the BigDye® Terminator v3.1 cycle sequencing kit chemistry. The assembled contigs were prepared by the CAP3 DNA assembly program. These newly obtained sequences were uploaded in the BLASTn suite to check whether they meet the threshold value of ≥97% for both the percent identity and query coverage. The sequences of the high‐fidelity amplicons were then deposited in the GenBank with the help of the Barcode Submission Tool with detailed source information and feature annotation.

### 2.3 Bioinformatic and statistical analyses

DNA sequences were translated in silico by the Translation Tool provided in the ExPASy portal to check for any pseudogenes and avoid sequencing errors. The composition of nucleotides was calculated on the CLC Workbench v7.7.1 and Mega X. The alignment of the amplicons was carried out in MUSCLE with the default specifications. Kimura two parameter (K2P) model was employed to calculate pairwise genetic distance at the level of species, genus, and family (Kimura, 1980). To estimate the K2P distances of the species with single sequence, we used at least 2–3 sequences of the same species available in GenBank submitted from Bangladesh or neighboring countries. Using this distance matrix, a neighbor‐joining (NJ) algorithm was implemented to generate the phylogenetic tree topology in Mega X with a bootstrapping value of 1,000 replications (Felsenstein, 1985; Saitou & Nei, 1987). Basic statistical analyses were done in Excel 2013 and the hypotheses testing (e.g., *t* test and *F* test) was done in RStudio‐1.2.5001.

## 3 RESULTS

### 3.1 General inference

A total of 748 tissue samples were collected from Bangladesh coast, among which 376 COI sequences were obtained (Table 1). Based on morphological and molecular identifications, these samples represented 185 species of 146 genera, 74 families, 21 orders and two classes (Table 1). Among the 185 species, 21 fishes were new records in Bangladesh. The length of all barcode sequences ranged from 477 to 683 bp, with an average of 652 bp and 89% of sequences were longer than 600 bp. No stop codon, insertion, or deletion was observed in any of the obtained sequences. The lack of stop codons in these sequences indicates that they are functional mitochondrial COI sequences, together with the fact that most of the amplified sequences was about 658 bp in length. Hence, it suggests that Nuclear DNA Sequences Originating from Mitochondrial DNA Sequences (NUMTs) were not sequenced, as vertebrate NUMTS are typically less than 600 bp (Zhang & Hewitt, 1996).

**TABLE 1:** List of marine fish species barcoded along with their GenBank accession numbers

SL No.	Order	Family	Species	No. of individual	GB Accession number
1	Anguilliformes	Congridae	*Conger japonicus*	1	MK995074
2		Muraenidae	*Strophidon sathete*	1	MH230989
3		Ophichthidae	*Ophichthus brevicaudatus*	1	MK995079
4	Aulopiformes	Synodontidae	*Saurida micropectoralis* ^a^	1	MH429316
5			*Harpadon nehereus*	1	MH087055
6	Batrachoidiformes	Batrachoididae	*Batrachomoeus trispinosus*	3	MN234104 MN234105 MN234107
7	Beloniformes	Belonidae	*Strongylura leiura*	4	MK926758 MN083098 MN013429 MT012664
8			*Strongylura strongylura*	1	MK995082
9		Hemiramphidae	*Hyporhamphus quoyi* ^a^	4	MN083114 MK988533 MK988540 MN200468
10			*Hemiramphus far* ^a^	1	MK995078
11			*Rhynchorhamphus malabaricus*	2	MF170953 MK988523
12		Zenarchopteridae	*Zenarchopterus ectuntio*	1	MK988518
13		Exocoetidae	*Xiphophorus maculatus*	2	MT012650 MT012672
14	Carcharhiniformes	Carcharhinidae	*Carcharhinus amboinensis*	1	MH230957
15			*Carcharhinus leucas*	1	MH230955
16			*Carcharhinus sorrah*	2	MH429287 MH429296
17			*Galeocerdo cuvier*	2	MN013428 MH429290
18			*Rhizoprionodon oligolinx*	5	MH311279 MH311281 MH311285 MH429294 MH429295
19			*Scoliodon laticaudus*	4	MH087056 MH087057 MH429292 MH230956
20		Sphyrnidae	*Sphyrna lewini*	3	MH429288 MH429289 MH230949
21	Clupeiformes	Clupeidae	*Anodontostoma chacunda*	2	MK878431 MH429338
22			*Escualosa thoracata*	1	MH429324
23			*Hilsa kelee*	4	KX657720 MN083113 MN083166 MN234106
24			*Sardinella longiceps*	5	KX988263 MK988539 MH311290 MH311291 MH429349
25			*Tenualosa ilisha*	2	KX657721 MH230965
26			*Tenualosa toli*	2	MH429339 KY124381
27			*Sardinella albella*	1	MK988545
28		Engraulidae	*Coilia dussumieri*	5	MN083117 MN171355 MK988524 MN200458 MH230984
29			*Coilia ramacarti*	3	MK926759 MN083109 MH311288
30			*Setipinna phasa*	2	MN083101 MH429325
31			*Setipinna tenuifilis*	1	MH429326
32			*Stolephorus dubiosus*	1	MN083107
33			*Stolephorus indicus*	3	MN083130 MK988535 MH311287
34			*Stolephorus waitei*	1	MH230967
35			*Thryssa hamiltonii*	2	MN083088 MH230969
36		Pristigasteridae	*Ilisha melastoma*	2	MN200469 MN200470
37			*Ilisha elongata*	5	MK926764 MK988525 MK988528 MK995085 MN200475
38			*Pellona ditchela*	1	MN083106
39			*Opisthopterus tardoore*	2	MN013414 MH311289
40	Elopiformes	Megalopidae	*Megalops cyprinoides*	2	MN171367 MN171368
41	Gadiformes	Bregmacerotidae	*Bregmaceros lanceolatus*	3	MN083116 MK988531 MN083100
42	Gonorynchiformes	Chanidae	*Chanos chanos*	1	MN083123
43	Mugiliformes	Mugilidae	*Liza tade*	3	MN083115 MK988537 MH429323
44			*Planiliza parmata*	4	MK988515 MH230960 MG550118 MK988538
45			*Chelon planiceps*	1	MK995096
46			*Mugil cephalus*	1	MK988536
47	Myliobatiformes	Dasyatidae	*Himantura gerrardi*	1	MH230945
48			*Himantura jenkinsii*	1	MH230946
49			*Himantura uarnacoides*	3	MH230951 MH230950 MH230953
50			*Himantura undulata*	1	MN013427
51			*Himantura walga*	8	MN013425 MN083136 MH429304 MH429305 MH429306 MH429310 MH882466 MH230948
52			*Neotrygon kuhlii*	2	MN013424 MN013426
53		Gymnuridae	*Gymnura poecilura*	3	MH230947 MH429307 MH429308
54		Myliobatidae	*Mobula mobular*	1	MH230952
55		Aetobatidae	*Aetobatus ocellatus*	1	MT012642
56	Orectolobiformes	Hemiscylliidae	*Chiloscyllium burmensis*	3	MN083134 MH429291 MH429293
57			*Chiloscyllium hasseltii*	2	MT021462 MT012673
58	Perciformes	Acropomatidae	*Acropoma japonicum*	1	MH311272
59		Ariommatidae	*Ariomma indicum*	1	MT012640
60		Carangidae	*Alepes djedaba*	2	MH429350 MT012666
61			*Alepes kleinii*	4	MN083120 MH429347 MN013415 MH230972
62			*Atropus atropos*	4	MH429313 MH429342 MT012674 MT012665
63			*Caranx sexfasciatus*	4	MN083119 MN083129 MK926754 MT012671
64			*Decapterus russelli*	1	MH429312
65			*Parastromateus niger*	3	MK926766 MH882455 MT012632
66			*Scomberoides commersonnianus*	1	MK926761
67			*Selar crumenophthalmus*	2	MH311273 MH882459
68			*Elagatis bipinnulata*	2	MK995097 MT012655
69			*Megalaspis cordyla*	4	MH429337 MH882458 MH230977 MH230976
70			*Scomberoides tol*	1	MH230981
71			*Alectis ciliaris*	1	MT012656
72		Cepolidae	*Acanthocepola indica*	1	MN083104
73		Coryphaenidae	*Coryphaena hippurus*	1	MN083097
74		Datnioididae	*Datnioides polota*	1	MN083087
75		Drepaneidae	*Drepane longimana*	1	MH429335
76		Ephippidae	*Ephippus orbis*	3	MH311274 MH429336 MH230987
77		Gerreidae	*Gerres filamentosus*	1	MH311269
78			*Pentaprion longimanus*	1	MH429311
79		Haemulidae	*Pomadasys argenteus*	1	MN083112
80			*Pomadasys kaakan* ^a^	1	MH311286
81			*Pomadasys maculatus*	4	MK988526 MK995076 MH429346 MH230986
82			*Diagramma picta*	1	MT012670
83		Istiophoridae	*Istiompax indica*	1	MH230959
84			*Istiophorus platypterus*	1	MT012639
85		Lactariidae	*Lactarius lactarius*	1	MH429314
86		Latidae	*Lates calcarifer*	3	MN171369 MG969518 MH087052
87		Labridae	*Halichoeres timorensis* ^a^	1	MT012644
88		Leiognathidae	*Nuchequula nuchalis*	1	MH230963
89			*Leiognathus equulus*	2	MT012636 MT012678
90			*Secutor ruconius*	3	MH311292 MH230964 MH429351
91		Lethrinidae	*Lethrinus crocineus* ^a^	1	MH429360
92		Lutjanidae	*Lutjanus johnii*	2	MK988516 MN171352
93			*Lutjanus lutjanus*	3	MH311266 MH311267 MT012648
94			*Lutjanus lunulatus*	1	MT012647
95			*Lutjanus lemniscatus*	1	MT012646
96			*Lutjanus fulvus*	1	MT012645
97			*Lutjanus indicus*	1	MT012652
98		Mullidae	*Upeneus sulphureus*	1	MH311268
99			*Upeneus supravittatus*	1	MT012634
100			*Upeneus moluccensis*	2	MN083158 MN083159
101			*Parupeneus indicus* ^a^	1	MT012679
102		Nemipteridae	*Nemipterus japonicus*	4	MH311271 MN083089 MH882457 MN200477
103			*Nemipterus mesoprion*	2	MH311270 MH230975
104			*Nemipterus randalli* ^a^	3	MN200456 MN200479 MN200480
105			*Scolopsis vosmeri*	2	MH311294 MH311295
106		Polynemidae	*Leptomelanosoma indicum*	3	MK995081 MH882456 MH230958
107			*Polynemus paradiseus*	5	MH087032 MH311275 MH311276 MH311282 MH230971
108			*Eleutheronema tetradactylum*	4	MN083108 MK988527 MN013416 MH230980
109			*Polydactylus sextarius*	1	MK995077
110		Priacanthidae	*Priacanthus prolixus*	1	MH230973
111		Rachycentridae	*Rachycentron canadum*	1	MN083099
112		Sciaenidae	*Argyrosomus regius* ^a^	1	MH429361
113			*Argyrosomus thorpei*	1	MG969524
114			*Chrysochir aureus*	3	MN083162 MH429341 MH230982
115			*Johnius carouna*	1	MH230988
116			*Johnius amblycephalus*	1	MK995084
117			*Otolithes ruber*	1	KY024208
118			*Pennahia anea*	2	KY024209 MT012635
119			*Protonibea diacanthus*	2	MN083125 MN083090
120			*Pterotolithus maculatus*	2	MN083096 MK988522
121		Scombridae	*Euthynnus affinis*	2	MK926763 MH230961
122			*Auxis rochei*	1	MT012638
123			*Rastrelliger brachysoma*	3	MH882460 MH311277 MH429343
124			*Scomberomorus guttatus*	4	MN083124 MK988517 MK988543 MH230970
125			*Scomberomorus plurilineatus*	2	MT012637 MH230978
126			*Scomberomorus commerson*	1	MT012641
127		Serranidae	*Cephalopholis formosa*	1	MH429359
128			*Cephalopholis boenak*	1	MH311293
129		Siganidae	*Siganas sutor* ^a^	3	MH882461 MH429334 MT012651
130			*Siganus fuscescens*	1	MH311264
131		Sillaginidae	*Sillaginopsis domina*	3	MH429318 MH429340 MH230968
132			*Sillago sihama*	1	MH429345
133		Sparidae	*Acanthopagrus butcheri*	4	MH230974 MK988519 MK995089 MN013431
134		Sphyraenidae	*Sphyraena chrysotaenia*	1	MH429315
135			*Sphyraena putnamae*	2	MN083102 MT012633
136		Menidae	*Mene maculata*	1	MT012631
137		Stromateidae	*Pampus argenteus*	6	MN083118 MK926765 MK926752 KX455908 MH429344 MH230962
138			*Pampus chinensis*	2	KX455907 MH882454
139		Terapontidae	*Terapon jarbua*	2	MK878430 MN013413
140			*Terapon theraps*	2	MN171358 MN171359
141		Trichiuridae	*Lepturacanthus savala*	4	MK995088 MN013417 MN013418 MH230979
142	Gobiiformes	Ambassidae	*Ambassis dussumieri*	1	MK995094
143		Gobiidae	*Acentrogobius nebulosus* ^a^	1	MN083110
144			*Boleophthalmus boddarti*	2	MN083126 MH429333
145			*Exyrias puntang* ^a^	1	MN083128
146			*Odontamblyopus rubicundus*	3	MH882462 MH882463 MH429321
147			*Parapocryptes serperaster*	1	MN083127
148			*Scartelaos histophorus*	5	MH087031 MK926760 MK988529 MN234102 MT012653
149			*Stigmatogobius sadanundio*	1	MK995090
150			*Oligolepis acutipennis* ^a^	1	MK988534
151			*Glossogobius giuris*	6	KT364791 MH087041 MK926756 MH429327 MT012669 MT012654
152			*Tridentiger barbatus* ^a^	1	MN083132
153			*Favonigobius gymnauchen* ^a^	2	MK995095 MN083121
154			*Pseudapocryptes elongatus*	4	MK926762 MK988530 MN013430 MN083091
155			*Trypauchen vagina*	3	MK926755 MH429320 MT012677
156			*Cryptocentrus cyanotaenia* ^a^	1	MT012659
157			*Istigobius ornatus* ^a^	1	MT012661
158	Pleuronectiformes	Cynoglossidae	*Cynoglossus oligolepis*	2	MH429298 MH429301
159			*Paraplagusia blochii* ^a^	3	MK995092 MH429300 MH429302
160		Paralichthyidae	*Pseudorhombus arsius*	3	MH311283 MH230944 MH429297
161			*Pseudorhombus natalensis*	1	MK995075
162		Soleidae	*Synaptura commersonnii*	2	MH311284 MH429299
163			*Zebrias altipinnis*	3	MH230943 MN083122 MH429303
164	Rhinopristiformes	Glaucostegidae	*Glaucostegus granulatus*	2	MH429309 MH230954
165	Scorpaeniformes	Platycephalidae	*Kumococius rodericensis*	2	MH311280 MK995080
166			*Platycephalus indicus*	1	MH429330
167			*Cociella crocodilus*	1	MT021463
168		Scorpaenidae	*Pterois russelii*	1	MH429332
169		Synanceiidae	*Minous monodactylus*	3	MH311265 MN083103 MH429331
170		Triglidae	*Lepidotrigla longimana* ^a^	3	MN083140 MN083141 MN083142
171			*Pterygotrigla hemisticta*	1	MN083139
172	Siluriformes	Ariidae	*Arius arius*	1	MK995087
173			*Osteogeneiosus militaris*	3	MH429317 MH429348 MH230983
174		Bagridae	*Rita rita*	2	MH087033 MH087043
175			*Mystus gulio*	4	KX455898 KX455905 MN083111 MK995086
176		Plotosidae	*Plotosus canius*	3	KX657716 MK995093 MN171370
177		Sisoridae	*Gagata gagata*	1	MH429322
178	Syngnathiformes	Syngnathidae	*Doryichthys boaja* ^a^	1	MK988541
179	Tetraodontiformes	Tetraodontidae	*Lagocephalus guentheri* ^a^	1	MH311278
180			*Lagocephalus lunaris*	1	MH429329
181			*Tetraodon fluviatilis*	1	MH429328
182			*Takifugu oblongus*	1	KT364766
183		Monocanthidae	*Aluterus monoceros*	1	
184	Torpediniformes	Narcinidae	*Narcine brunnea*	4	MH882464 MH882465 MN083105 MH429319
185		Narcinidae	*Narcine maculata*	1	MN083137

^a^ Species of new records.

### 3.2 Elasmobranchii (Sharks and rays)

A total of 52 samples were sequenced belonging to 5 order, 8 families, 13 genera, and 21 species (Table 1). Among these 21 species, one species is listed as critically endangered (CR), four as endangered (EN), four as vulnerable (VU), six as near threatened (NT), one as least concern (LC), three not evaluated (NE) and the remaining two species are data deficient (DD) in the IUCN Red List (IUCN, 2020). The sequence analysis indicated the average nucleotide frequencies to be A: 25.90%, T: 32.70%, G: 16.20%, and C: 25.20%. The base composition analysis for the COI sequence showed that the average percent T content was the highest and the average percent G content was the lowest; the AT content (58.60%) was higher than the GC content (41.40%). The GC contents at the first, second, and third codon positions for the Elasmobranchii were 53.30%, 43.60%, and 27.70%, respectively. At the first codon position, the usage of T (20.00%) was the lowest, and the usages of the other bases were 23.50%, 27.40%, and 29.50% for C, A, and G, respectively. At the second codon position, the content of T (42.00%) was highest, and the percentage of the other bases were 29.40%, 14.50%, and 14.20% for C, A, and G, respectively. At the third codon position, the base usage was T: 37.00%, C: 22.80%, A: 35.70%, and G: 4.90%; the G content being the lowest, exhibited a clear pattern of anti‐G bias.

**FIGURE 2:**
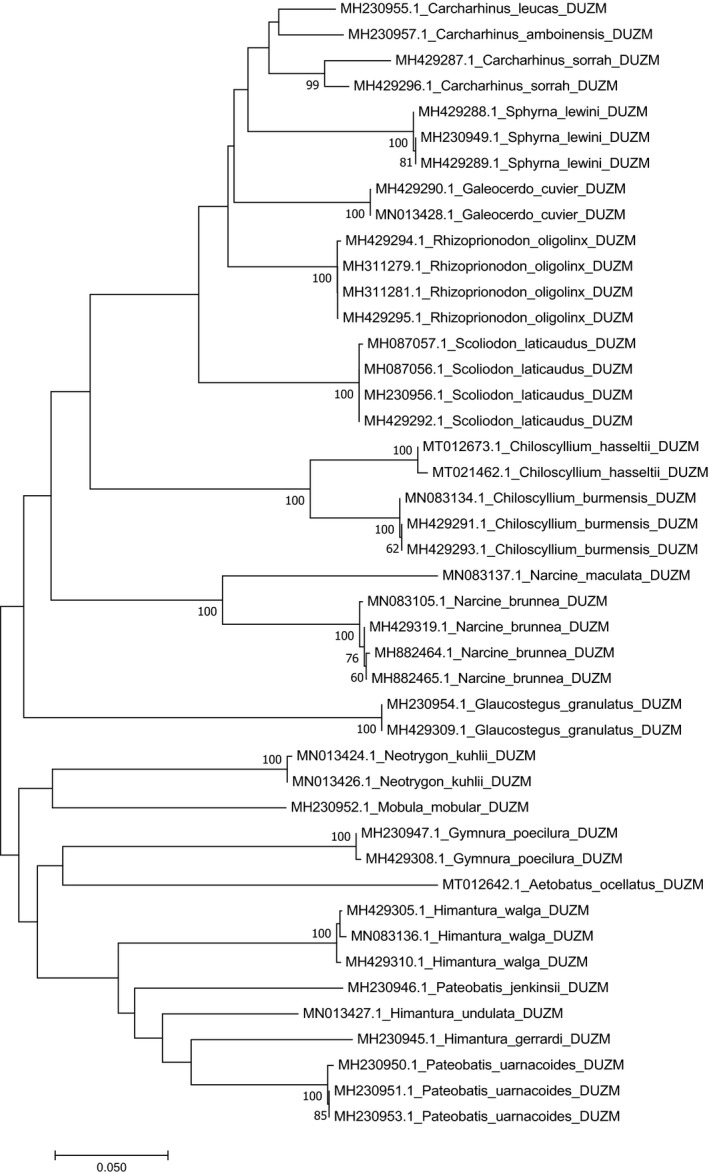
Neighbor‐joining (NJ) tree of 21 species of Elasmobranchii using K2P distances

The K2P genetic distances within each taxonomic level are summarized in Table 2. The average genetic distance within species, genus, family, and order were 1.20 ± 0.0007%, 6.07 ± 0.014%, 11.08 ± 0.018%, and 14.68 ± 0.04%, respectively. The NJ tree clearly distinguished all the species and the species belonging to 8 families were represented by eight distinct clades (Figure 2).

**TABLE 2:** Genetic divergence (%K2P distance) of Elasmobranchii within various taxonomic levels

Level	Sample size	Mean	Minimum	Maximum	*SE*
Species	21	1.20	0.00	2.90	0.0007
Genus	13	6.07	0.36	14.20	0.014
Family	8	11.08	2.50	18.60	0.018
Order	5	14.68	10.57	22.81	0.04

### 3.3 Actinopterygii (Ray‐finned fishes)

A total of 324 sequences were generated belonging to 16 orders, 66 families, 133 genera and 164 species (Table 1). Among the studied 164 species of ray‐finned fishes, two are listed as endangered (EN), two as vulnerable (VU), four as near threatened (NT), 108 as least concern (LC), 12 as data deficient (DD) and the rest 36 are not evaluated (NE) in the IUCN global Red List (IUCN, 2020). No such assessment has been conducted yet for marine fishes of Bangladesh. We assume that some of these commercially important species are indiscriminately overexploiting and threatened by extinction from this region. So, the country now needs to assess its national red list status of marine fishes to enforce conservation and management strategy.

The sequence analysis revealed average nucleotide frequencies to be A: 23.9%, T: 29.6%, G: 18.3%, and C: 28.2%. The base composition analysis for the COI sequence showed that the average T content was the highest and the average G content was the lowest; the AT content (53.50%) was higher than the GC content (46.50%). The GC contents at the first, second, and third codon positions for the fish species were 54.62%, 43.66%, and 38.04%, respectively. The pattern of %GC content at different codons was 1st> 2nd> 3rd (*p*‐value < 0.005) The K2P genetic distances within each taxonomic level are summarized in Table 3. The average genetic distance within species, genus, family and order were 0.40 ± 0.002%, 6.36 ± 0.008%, 14.10 ± 0.01% and 24.07 ± 0.02%, respectively (Table 3).

**TABLE 3:** Genetic divergence (% K2P distance) of Teleosts within various taxonomic levels

Level	Sample size	Mean	Minimum	Maximum	*SE*
Species	164	0.40	0	1.88	0.002
Genus	132	6.36	0.02	28.57	0.008
Family	66	14.10	1.30	51.10	0.01
Order	15	24.07	18.46	29.96	0.02

**FIGURE 3:**
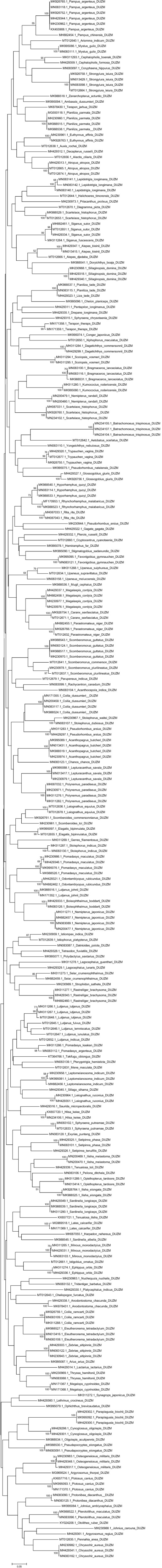
Neighbor‐joining (NJ) tree of 164 teleost fish species, using K2P distances

The NJ tree of all generated sequences included 164 species is provided in Figure 3. Most of the specimens of the same species were clustered together, which reflected the prior taxonomic assignment based on morphology. No taxonomic deviation was detected at the species level, indicating that the majority of the examined species could be authenticated by the barcode approach.

#### 3.3.1 Order Clupeiformes

This order includes many of the most important forage and food fish. A total of 45 samples were sequenced belonging to three families, 12 genera and 19 species. Among the three families Clupeidae is the most valuable family where a single species *Tenualosa ilisha* contributes over 12% of total fish production of the country (DoF, 2018). The overall mean nucleotide base frequencies observed for these sequences were T: 28.60%, C: 27.80%, A: 24.20%, and G: 19.40%. The AT content (52.80%) was higher than the GC content (47.20%). The GC contents at the first, second, and third codon positions were 49.10%, 48.20%, and 44.40%, respectively. The K2P distances of the COI sequence within species, genera and family were 1.81, 6.55, and 13.41, respectively (Table 3). The NJ tree clearly distinguished all the species. The species belonging to family Clupeidae, Engraulidae, and Pristigasteridae were represented by three distinct clades.

#### 3.3.2 Order Perciformes

Perciformes is the most dominant order among the marine fishes of Bangladesh and contributes to over 45% of total exploited species. A total of 161 samples were sequenced belonging to 33 families, 63 genera, and 84 species. Among the 33 families, Carangidae was the most dominant one followed by Sciaenidae and Polynemidae. The overall mean nucleotide base frequencies observed for these sequences were T: 29.60%, C: 28.50%, A: 23.40% and G: 18.50%. The AT content (53.00%) was higher than the GC content (47.00%). The GC contents at the first, second, and third codon positions were 56.80%, 42.70%, and 41.50%, respectively. The K2P distances of the COI sequence within species, genera and family were 0.58, 6.26, and 10.60, respectively. In the NJ tree, most of the specimens belonging to the same species were clustered together bolstering the prior taxonomic assignment based on morphology.

#### 3.3.3 Order Gobiiformes

Gobiiformes has recently been segregated from the Perciformes as a new order (Nelson et al., 2016). The average base composition was T: 30.1%, C: 27.4%, A: 24.4% and G: 18.1% for the 16 species of Gobiiformes under the two families. The AT content (54.50%) was higher than the GC content (45.50%). The average GC content in 1st, 2nd, and 3rd codon was 55.2%, 44.8%, and 37.7%, respectively which follow 1st> 2nd> 3rd codon. The K2P distances of the COI sequence within species, family were 0.59% and 13.22%, respectively.

#### 3.3.4 Order Siluriformes

A total of 14 samples were sequenced belonging to four families, six genera, and six species. The overall mean nucleotide base frequencies observed for these sequences were T: 29.30%, C: 28.10%, A: 24.80%, and G: 17.70%. The AT content (54.10%) was higher than the GC content (45.90%). The GC contents at the first, second, and third codon positions were 56.70%, 42.70%, and 38.10%, respectively. The K2P distances of the COI sequence within species and between species were 0.14 and 26.62, respectively.

#### 3.3.5 Order Pleuronectiformes

Fourteen samples were sequenced belonging to three families, five genera, and six species. The overall mean nucleotide base frequencies observed for these sequences were T: 30.80%, C: 26.70%, A: 24.10% and G: 18.30%. The AT content (54.90%) was higher than the GC content (45.10%). The GC contents at the first, second, and third codon positions were 55.50%, 42.10%, and 37.60%, respectively. The K2P distances of the COI sequences within species and family were 0.36 and 17.65, respectively.

#### 3.3.6 Order Beloniformes

Fifteen samples were sequenced belonging to four families, six genera and seven species. The overall mean nucleotide base frequencies observed for these sequences were T: 32.40%, C: 25.90%, A: 24.90%, and G: 16.80%. The AT content (57.30%) was higher than the GC content (42.70%). The GC contents at the first, second, and third codon positions were 55.10%, 42.60%, and 30.60%, respectively. The K2P distances of the COI sequence within species, genera, and family were 0.24, 4.77, and 9.67, respectively.

## 4 DISCUSSION

DNA barcoding has been adopted as a global bio‐scanner to provide an efficient molecular technique for species‐specific identification using the partial sequence of the mitochondrial COI gene. It is evident from more than a decade of studies (Chang et al., 2017; Hubert et al., 2008; Lakra et al., 2011; Thu et al., 2019; Ward et al., 2005; Zhang, 2011) that the barcoding can discriminate marine fish species inhabiting different geographic regions, including Australia, Canada, China, India, Taiwan, and Vietnam. Here, we have profiled the barcode of marine fishes collected from the coast of Bangladesh and demonstrated the efficacy of barcoding to identify these, exploiting the partial sequence of mitochondrial COI genes. Barcodes were generated for 185 species of Elasmobranchii and Actinopterygii from Bangladesh belonging to 146 genera and 74 families and 21 orders (Table 1). We observed no insertions/ deletions or codon stops after translating the nucleotide sequences, supporting the view that all of the amplified sequences denote functional mitochondrial COI sequences. Moreover, the average length of the amplified sequences was larger than 650bp, the limit typically observed for nuclear DNA sequences originating from mtDNA (NUMTs; Gunbin et al., 2017). All of these species were differentiable based on the individual COI barcodes. Hence, this study has strongly validated the efficiency of COI barcodes for identifying fish species.

Within the Elasmobranchii, a total of 12 rays and nine sharks species were confirmed through barcoding. The overall AT and GC content was 58.60% and 41.40%, respectively. But the mean GC content of the 11 barcoded ray species was higher than the 8 shark species (43.67% vs. 38.59%). This was largely due to the GC variation in the 3rd codon position (33.59% vs. 20.38%).

In this study, the COI barcode sequences for 164 ray‐finned fish species were successfully amplified (Table 1; Figure 3). At least 21 new records have been confirmed in this study based on morphometric and molecular approach (Table 1). Among them, seven species belonged to the family Gobiidae and most of them are relatively small in size, inhabit estuarine and near‐shore area. Taking into account the marine ichthyofaunal diversity in the neighboring waters of India and Myanmar (Froese & Pauly, 2019; Gopi & Mishra, 2015), it could be presumed that an intensive faunal survey and authentic taxonomic identification might explore much more new records or species from Bangladesh waters.

The base composition analysis of the COI sequences revealed AT content (53.50%) to be higher than GC content (46.50%), similar to the pattern observed in Australian (Ward et al., 2005), Canadian (Steinke et al., 2009) and Cuban fish species (Lara et al., 2010). The GC contents in the first, second, and third codon positions were 54.62%, 43.66%, and 38.04%, respectively. At the first codon position, the usage of G (20.00%) was the lowest, and the usages of the other bases were 28.60%, 27.90%, and 27.00% for C, A, and T, respectively. At the second codon position, the content of T (32.00%) was highest, and the contents of the other bases were C: 29.50%, A: 20.20% and G: 18.30. At the third codon position, the base usage was T: 30.00%, C: 26.40%, A: 23.50% and G: 16.50%. There was a significantly higher overall GC content in the 164 species of bony fish compared to the 21 species of sharks and rays (46.50% vs. 41.40% with a *p*‐value ˂ 0.005). This difference was attributable to the GC content at the 2nd (47.80% versus 43.60%) and, especially, the 3rd codon base (42.80% versus 27.70%). The pattern of %GC content at different codons for all these fishes was invariably 1st > 2nd > 3rd (*p*‐value < 0.005) and for Pleuronectiformes 1st > 2nd > 3rd (*p*‐value < 0.05, *n* = 6).

**FIGURE 4:**
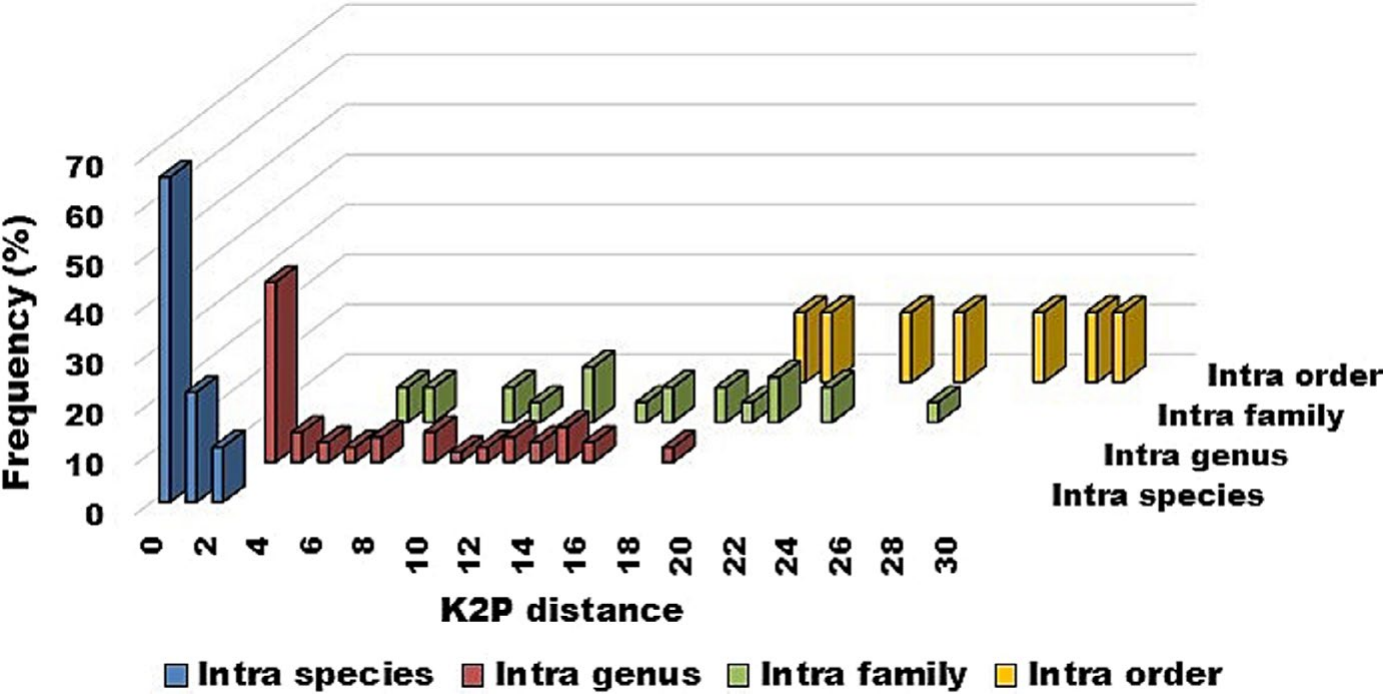
Distribution of K2P distances (percentage) within different taxonomic categories

Kimura 2‐parameter distance values of 6.36 ± 0.008%, 14.10 ± 0.01%, and 24.07 ± 0.02% were obtained for within genus, within family and within order, respectively (Table 3; Figure 4). Consistent with previously published fish barcoding data, pairwise genetic distance values were increasing at higher taxonomic levels. This increase in the genetic distance through the higher taxonomic levels supports the significant change in genetic divergence at the species boundaries (Hubert et al., 2008; Lakra et al., 2011).

In this study, the average within species K2P distance was 0.40%, compared with 6.36% for within genera. The mean interspecific distance was found to be 16‐fold higher than the mean intraspecific distance. More than 13.9‐fold difference was observed in the marine fishes commonly encountered in the Canadian Atlantic (Steinke et al., 2009), Indian (Lakra et al., 2011) and Australian marine fishes (Ward et al., 2005). This result corresponds to the DNA barcoding principle that interspecific divergence sufficiently outscores intraspecific divergence.

The accuracy of species identification through DNA barcoding mostly depends on both interspecific and intraspecific divergence. In our study, the average genetic distance within species was found 0.40 ± 0.002%. Mean intraspecific genetic distance was calculated as <1% in previous studies; Hubert et al. (2008) found 0.30% (0%–7.42%) for 194 fish species from Canadian ichthyofauna; Ward et al. (2005) 0.39% (0%–14.08%) for 207 marine fish species from Australia; Thu et al. (2019) 0.34% for 458 ray‐finned species in Vietnam and Bingpeng et al. (2018) found 0.21% for 85 genera in Taiwan strait (Table 4).

**TABLE 4:** Summary of genetic divergences (% K2P) of marine fishes within various taxonomic levels based on COI sequences from different geographic regions

Country	Species	Genus	Family	Order	References
Australia	0.39	9.93	15.46	22.18	Ward et al. (2005)
India	0.30	6.60	9.91	16.00	Lakra et al. (2011)
South China Sea	0.18	13.55	19.65	24.05	Zhang (2011)
Rongcheng Bay, China	0.21	5.28	21.30	23.63	Wang et al. (2018)
Vietnam	0.34	12.14	17.30	21.42	Thu et al. (2019)
Taiwan	0.21	6.50	23.70	25.60	Bingpeng et al. (2018)
Bangladesh	0.40	6.36	14.10	24.07	Present study Ahmed et al. (2021)

Phylogenetic relationship of barcoded species of Elasmobranchii and Actinopterygii were shown in separate NJ tree (Figures 2 and 3). Each species was associated with a specific DNA barcode cluster and the relationship among these species was clearly revealed. Closer species in terms of genetic divergence were clustered at the same nodes and the distance between the terminal branches of the NJ tree widened as they got more distinct. No cryptic diversity was recorded especially in this biodiversity hotspot area. Perhaps, the reason was number of replicates for each species was relatively small in this study. Our extended research on rest of the marine species with large sample sizes from different locations might reveal the potential cryptic biodiversity among marine fishes of Bangladesh in future.

Present study suggests that DNA barcoding has been successful in identifying and discriminating the vast majority of marine ichthyofauna. The DNA barcoding method has been proven to be an effective tool for species identification, particularly with specimens that are damaged, incomplete, or consisting of several morphologically distinct stages (Bingpeng et al., 2018; Pečnikar & Buzan, 2014). Nevertheless, DNA barcoding also has its limitations. In some cases, related species may present identical sequences making DNA barcodes useless for species discrimination. Therefore, DNA barcoding can serve as a complementary tool for species identification, though it cannot replace the traditional morpho‐taxonomy. Through this study, a reliable DNA barcode reference library for the marine fish in the Bay of Bengal, Bangladesh has been established, which could be used to assign fish species by screening sequences against it in the future. We hope this would appreciably contribute to achieving better monitoring, conservation, and management of fisheries in this overexploited region.

## CONFLICT OF INTEREST

The authors declare no conflict of interest.

## AUTHORS’ CONTRIBUTIONS

Ahmed MS initiated the project, acquired funding, managed project administration, specimen curation, and internal reports, performed the taxonomic analyses, and prepared the whole manuscript. Datta SK and Saha T performed field collection, generated and analyzed molecular sequences, curated tissue samples, and participated in the writing of the final manuscript. Hossain Z contributed laboratory analyses, participated in generation of sequences and made contribution to the manuscript. All authors reviewed the manuscript.

## ETHICAL APPROVAL

Collection of fish samples were conducted under a permit to the University of Dhaka, and approval of the Ethical Review Committee, Faculty of Biological Sciences, University of Dhaka.

## Data Availability

DNA sequences: The DNA barcoding data can be retrieved from the NCBI GenBank (https://www.ncbi.nlm.nih.gov/) as it has open public access. GenBank Accession Nos for each individual are shown in Table 1. Voucher specimens: All the voucher specimens with their respective Voucher ID are kept at the Museum of Department of Zoology, University of Dhaka, Bangladesh, and have public access with permission.
